# Screening MT1-MMP Activity and Inhibition in Three-Dimensional Tumor Spheroids

**DOI:** 10.3390/biomedicines11020562

**Published:** 2023-02-15

**Authors:** Anna M. Knapinska, Gary Drotleff, Cedric Chai, Destiny Twohill, Alexa Ernce, Dorota Tokmina-Roszyk, Isabella Grande, Michelle Rodriguez, Brad Larson, Gregg B. Fields

**Affiliations:** 1 Alphazyme, Jupiter, FL 33458, USA; 2 Institute for Human Health & Disease Intervention (I-HEALTH), Florida Atlantic University, Jupiter, FL 33458, USA; 3 Agilent Technologies, Raleigh, NC 27606, USA

**Keywords:** membrane type 1 matrix metalloproteinase, glioblastoma, spheroid, cell-based high-throughput screening, triple helix, collagen, FRET assay

## Abstract

Membrane type 1 matrix metalloproteinase (MT1-MMP) has been shown to be crucial for tumor angiogenesis, invasion, and metastasis, and thus MT1-MMP is a high priority target for potential cancer therapies. To properly evaluate MT1-MMP inhibitors, a screening protocol is desired by which enzyme activity can be quantified in a tumor microenvironment-like model system. In the present study, we applied a fluorogenic, collagen model triple-helical substrate to quantify MT1-MMP activity for tumor spheroids embedded in a collagen hydrogel. The substrate was designed to be MT1-MMP selective and to possess fluorescent properties compatible with cell-based assays. The proteolysis of the substrate correlated to glioma spheroid invasion. In turn, the application of either small molecule or protein-based MMP inhibitors reduced proteolytic activity and glioma spheroid invasion. The presence of MT1-MMP in glioma spheroids was confirmed by western blotting. Thus, spheroid invasion was dependent on MT1-MMP activity, and inhibitors of MT1-MMP and invasion could be conveniently screened in a high-throughput format. The combination of the fluorogenic, triple-helical substrate, the three-dimensional tumor spheroids embedded in collagen, and Hit-Pick software resulted in an easily adaptable in vivo-like tumor microenvironment for rapidly processing inhibitor potential for anti-cancer use.

## 1. Introduction

Malignant gliomas, including glioblastoma multiforme (GBM) and astrocytomas, are the most common primary brain tumors in the United States [1] and make up 78% of malignant brain tumors [2]. Although relatively rare, particularly in the United States, malignant brain tumors exhibit a disproportionate cancer mortality due to their high fatality rates [3], where, on average, only one-third of individuals survive at least five years after diagnosis [4]. Membrane type 1 matrix metalloproteinase (MT1-MMP, also known as MMP-14) has been shown to be crucial for the progression, invasion, migration, and angiogenesis of tumors [5,6,7]. MT1-MMP belongs to a subset of zinc-dependent membrane-anchored MMPs able to degrade the basement membrane and proteins of the extracellular matrix (ECM) [8], cell adhesion molecules, cytokines, growth factors, and receptors [9]. Its expression also correlates with tumor grade and is associated with reduced survival in glioma patients [10,11,12,13,14]. Therefore, many studies have highlighted the potential of MT1-MMP as a therapeutic target in a variety of cancers [15,16].

Invasion is considered one of the main hallmarks of cancer [17,18] and is the result of the continuous interaction between tumor cells and the surrounding microenvironment [17,18,19]. When glioma cells invade through the brain parenchyma, they establish contact with molecules of the ECM and components of the basement membrane. As part of this process, surface proteases, such as MT-MMPs, are recruited at the focal contacts with the ECM where they either directly or indirectly (through the activation of soluble MMPs) degrade and remodel the surrounding ECM, favoring invasion [20]. For many years, the simplest model to study cell motility, especially with respect to cell-ECM interactions, consisted of culturing cells in a two-dimensional (2D) monolayer on glass, plastic slides, or microplates [21]. ECM components were used either as a coating or solubilized in the medium, and the assay involved the observation of motility [21]. Despite the simplicity of the model, there were numerous limitations [21]; specifically, the assay monitored motility and not invasion [22]. It also did not take into account the fact that cells in monolayers behave differently than cells cultured in a three-dimensional (3D) manner [23,24]. Because of this limitation, newer, more advanced cell models have been developed to better mimic the in vivo cell environment. A particular model showing promise incorporates round-bottom spheroid microplates where tumor specific primary cells, or cultured cell lines, are added to plate wells. Following aggregation, the 3D tumor models are embedded in a hydrogel, such as collagen, Matrigel, etc., and allowed to invade into the ECM. This type of model allows for more complex development that more closely mimics tumor development [25,26] and gives rise to tumors resembling the in vivo invasion pattern of the tumor of origin [25,26]. It has also been demonstrated that this 3D invasion model led to the increased production of matrix degrading enzymes, such as MMPs [27,28], and was more predictive of therapy response [26,29].

In the present study, multiple glioma cell lines were formed into tumor spheroids, followed by the addition of type I collagen. The FAM-fTHP-9 substrate was utilized to monitor MT1-MMP activity. FAM-fTHP-9 is a fluorescently labeled collagen model synthetic triple-helical peptide substrate designed to be preferentially targeted and degraded by MT1-MMP [30]. A variety of known MT1-MMP inhibitors were then added to the wells to potentially inhibit the invasion of the spheroids. During the procedure, all control and test wells were read and the signal from the cleaved substrate was quantified. Uninhibited invasion within positive control wells exhibited high fluorescence values. Therefore, a “Hit Pick” criteria was established wherein any wells containing test molecules that were statistically lower in fluorescent signal than the average from the corresponding positive control, and therefore were expected to exhibit inhibited invasion, were imaged. Those wells that were not significantly lower were not imaged. To evaluate the general utility of this approach, melanoma spheroids were also examined.

## 2. Materials and Methods

A-172 glioblastoma cells (catalog # CRL-1620), H4 astrocytoma cells (catalog # HTB-148), SW 1088 astrocytoma cells (catalog # HTB-12), U-87 glioblastoma cells (catalog # HTB-14), WM-266-4 metastatic melanoma cells (catalog # CRL-1676), and WM115 primary melanoma cells (catalog # CRL-1675) were obtained from ATCC (Manassas, VA, USA). Type I collagen solution from rat tail (catalog # C3867), collagenase D (catalog # COLLD-RO), and marimastat (catalog # M2699) were purchased from Sigma-Aldrich (St. Louis, MO, USA). Hyclone Fetal Bovine Serum (catalog # SH3007103) was sourced from GE Healthcare LifeSciences (Piscataway, NJ, USA). Penicillin-streptomycin (100×) (catalog # 15140-122), collagenase D (catalog # NC1578589), and Hepes, 1 M (catalog # 15630-080) were purchased from Thermo Fisher Scientific (Waltham, MA, USA). Recombinant MMP-14 (catalog # 918-MP), aprotinin (catalog # 4139), and recombinant human TIMP-2, CF (catalog # 971-TM) were purchased from R&D Systems (Minneapolis, MN, USA). 96-Well Black/clear round bottom ultra-low attachment spheroid microplates (catalog # 4515) were purchased from Corning Life Sciences (Corning, NY, USA). Cell culture materials were purchased from Fischer Scientific (Pittsburgh, PA, USA). FAM-fTHP-9 (sequence Gly-Pro-Hyp)_5_-Gly-Pro-Lys(Fam)-Gly-Pro-Gln-Gly~Cys(Mob)-Arg-Gly-Gln-Lys(Dabycl)-Gly-Val-Arg-(Gly-Pro-Hyp)_5_-NH_2_) was synthesized and characterized as previously described [31].

The concentration of FAM-fTHP-9 was determined using a Nanodrop (Thermo Fisher Scientific) with an absorbance measuring at λ = 493 nm. The substrate was subsequently prepared as 40 and 80 μM stock solutions in Tris Salt buffer (TSB) (50 mM Tris, 100 mM NaCl, 10 mM CaCl_2_, 0.05% Brij-35, pH 7.5).

A-172, H4, and WM115 were incubated in a 5% CO_2_ incubator at 37 °C and were maintained in tissue culture-treated T-75 flasks (Thermo Fisher Scientific) in Dulbecco’s Modification of Eagle’s Medium (DMEM) (Fisher Scientific) supplemented with heat-inactivated 1% fetal bovine serum (FBS), 1% penicillin-streptomycin, and sodium pyruvate (1×). U-87 and WM266 were incubated similarly and maintained in Eagle’s Minimal Essential Medium (EMEM) (Fisher Scientific) supplemented with heat-inactivated 1% FBS, 1% penicillin-streptomycin, and sodium pyruvate (1×). Heat inactivation was performed at 56 °C for 30 min in a water bath with occasional shaking [32]. SW1088 was incubated in a 0% CO_2_ incubator at 37 °C and maintained in L-15 media. The cell lines were split or passaged between 70–90% confluency. Cell passaging was preformed at regular intervals of passaging every 4 d. Cells were detached by washing with 1× phosphate-buffered saline solution (FBS), then PBS was removed from the flask. A dissociating agent, 1× TrypLE Express Enzyme (Thermo Fisher Scientific), was added to the flask and incubated for 3–5 min to detach the cells. TrypLE was deactivated by adding a 1:1 ratio of complete DMEM to the flask. The cell suspension was then diluted to each cell line’s respective subcultivation ratio (as recommended by ATCC) with completed media and added to a new cell culture flask.

Cell counts were obtained from cell suspensions using Trypan Blue (Thermo Fisher Scientific). A combination of Trypan Blue and the cell suspension were mixed in a 1:1 ratio and then transferred into a cell counting chamber slide for the cells to be counted using a Countess™ II Automated Cell Counter (Thermo Fisher Scientific). Each cell line suspension was prepared at a concentration of 50,000 cells/mL using the detached cells and complete DMEM. The prepared suspensions were then seeded into Corning 4515 96-Well Ultra-Low Binding, U-Shaped Bottom microplates at 200 μL/well in order to form a single spheroid per well. The spheroids were observed after the cells were incubated for at least 72 h in 5% CO_2_ at 37 °C. After individual spheroids were formed, 150 μL of media was removed from each well and the individual spheroids were then embedded in 96-well microplates containing 150 μL per well of Advanced Biomatrix PURECOL EZ GEL solution of type I collagen (where this solution contained 750 mL of stock collagen solution, 250 mL of cell type-appropriate complete media, and 30 mL of 1 M HEPES). The plate was then incubated for at least 1 h to allow the collagen to congeal. Pictures were taken at time zero and at several time points to display the growing presence and amount of invadopodia over time.

A test for fluorescence intensity was conducted to confirm that one can detect FAM-fTHP-9 cleavage in spheroids embedded in type I collagen. Two broad spectrum MMP inhibitors (marimastat and aprotinin) were added to wells as a treatment. Marimastat was added at a final concentration of 400 μM, and aprotinin was added at a final concentration of 4 μM. Wells treated with these inhibitors were allowed to incubate for 1 h in 5% CO_2_ at 37 °C. After incubation, FAM-fTHP-9 was thoroughly mixed with type I collagen before embedding the spheroids in the collagen. FAM-fTHP-9 was prepared in two concentrations: 80 μM of the substrate was added to wells containing spheroids embedded in collagen, while 40 μM of substrate was added to wells containing spheroids in media only. Fluorescence was monitored continuously on a Cytation 5 Microplate Reader (BioTek, Winooski, VT, USA), using λ_excitation_ = 485 nm and λ_emission_ = 528 nm, at 37 °C for 36 h. Measurements were reported in relative fluorescence units (RFUs).

A-172 glioma spheroids and WM115 melanoma spheroids were treated with 150 μL (diluted in a 1:3 ratio with PBS) of type I collagen per well and allowed to incubate for 72 h in a 5% CO_2_ incubator. The spheroids pretreated with collagen were then exposed to collagenase D, 100 μL/well. The plate was then incubated for 2 h in a Cytation 5 plate reader (BioTek) that provided orbital shaking in 15 min intervals.

Cell viability was determined by CellTiter-Glo^®^ 3D Cell Viability Assay (Promega; Maddison, WI, USA) as per the manufacturer’s instructions. For western blot analysis, lysate was collected as follows. Seventy-two hours after collagen embedding, 100 μL of collagenase was added to each collagen-containing well for 2 h at 37 °C with orbital shaking on the Citation 5. Six spheroids were then pooled together and rinsed with phosphate-buffered saline (PBS). For cell lysis, the buffer consisted of 10 mM Tris•HCl, 150 mM NaCl, 1 mM EDTA, and 1% Triton X-114 supplemented with 1 mM PMSF (Sigma-Aldrich) and 1X Protease Inhibitor Cocktail III (Thermo Scientific). Lysis was achieved at a concentration of 500,000 cells/100 μL incubated at 4 °C for 1 h with mild agitation. The resulting supernatant was used for western blot analysis.

Western blotting was performed as follows. The total protein concentration was determined using the Bradford method. Twenty μg of each sample was resolved in a 10% SDS-polyacrylamide gel and transferred to a nitrocellulose membrane (Thermo Scientific). The total protein content present on the membrane was determined using the Revert 700 Total Protein Stain for western blot normalization (LICOR, Lincoln, NE, USA). The membranes were blocked with PBS containing 5% nonfat dried milk and 0.05% Tween 20 and then incubated overnight at 4 °C with either a recombinant monoclonal rabbit anti-human MT1-MMP antibody (Abcam, Cambridge, UK) or a polyclonal rabbit anti-human MT1-MMP antibody (Sigma-Aldrich) at a concentration of 1:1000. The membrane was then washed with PBST followed by 1.5 h incubation with IRDye 800CW donkey anti-rabbit IgG secondary antibody (LICOR). Afterwards, the wash step was repeated and the image acquired using LICOR’s Odyssey imaging system.

Cells were harvested in a final concentration of 5.0 × 10^4^ cells/mL for each cell type in complete DMEM. After dispensing 200 μL of cell suspension into appropriate microplate wells, the microplates were incubated at 37 °C/5% CO_2_ for 72 to 96 h, depending on cell type, to allow cells to aggregate into tumoroids. Upon completion of tumoroid formation, 80–90% of complete medium was manually removed from each well, and the tumoroid plate placed on ice in a refrigerator for 5 min to cool the cells. Collagen matrix was then thawed on ice. Once thawed, a working solution was then created such that every 1 mL contained 750 μL of stock collagen solution, 250 μL of complete cell type appropriate complete media, and 30 μL of 1 M HEPES. With the plate still on ice, 150 μL of collagen working solution was added to each well of the spheroid microplates. The microplate was centrifuged at 300× *g* for 5 min in a swinging bucket centrifuge that was previously set to 4 °C for tumoroid positioning, then transferred to a 37 °C/5% CO_2_ incubator for one hour to initiate gel formation.

Following gel formation, microplates were removed from the incubator and an equal volume of cell specific complete media added to the wells, with or without test inhibitors. The final concentrations of inhibitors were 400 μM marimastat, 4 μM aprotinin, and 30 μM TIMP-2. The plates were once again placed back into the incubator for 1 h. Finally, FAM-fTHP-9 was added to each well at a final concentration of 5 μM. Upon the addition of FAM-fTHP-9, spheroid plates used to test the ability to kinetically track fluorescent substrate signal and tumoroid invasion were placed into the Cytation 5. A discontinuous kinetic procedure was created to both detect the green fluorescent signal from each well using the Cytation 5′s variable bandwith monochromators in addition to capturing tumoroid images using the brightfield transmitted light imaging channel. Reading and imaging were carried out every 6 h over a 48-h time period.

Following the addition of inhibitor molecules and substrate, as previously described, spheroid plates were placed into a tissue culture incubator for an additional 48 h. At the end of the incubation period, the plates were transferred into the Cytation 5 to perform the Hit Pick experiment [33]. The automated procedure consisted of three steps. In the first step, a fluorescence intensity read was carried out on all appropriate wells of the plate for the cell type being tested with the Cytation 5′s monochromators. Settings for the monochromators were the same as those used for the kinetic reading and imaging experiments. By selecting “Define statistic” in the Hit Pick step window, a statistically defined criteria was then determined to trigger test well imaging. The average and standard deviation of the signal from untreated, positive control wells, which should exhibit the highest amount of FAM-fTHP-9 cleavage and the therefore highest signal for each cell type, were automatically calculated by Gen5. The criteria were then set such that that the signal from inhibitor test wells had to be greater than two standard deviations below the average signal from the control wells. If the signal was lower than the cutoff criteria, the test well was imaged. If the signal did not meet the criteria, the well was left unimaged. In the final part of the Hit Pick procedure, an imaging step was carried out to discern the level of tumoroid invasion following compound incubation using the brightfield imaging channel. The same imaging settings were used as those for the kinetic reading and imaging experiments.

Individual image tiles from each z-plane captured using the one-by-two montage were then stitched together. A single image projection was then created from the 16 slice stitched z-stack. The focus stacking method was chosen, which automatically selects the most in-focus pixel from each image in the stack for inclusion in the final projection. This allows for a most accurate analysis to be carried out on each invading tumoroid at each timepoint. A signal background removal step was then applied to the stitched, projected images. This served to even out the background brightfield signal and increase the accuracy in terms of cellular analysis mask placement. Following image processing, primary cellular analysis criteria were applied using the brightfield channel to automatically place object masks around the entire invading structure in each final image.

Statistical analysis was performed using GraphPad Prism 8 (GraphPad Software, Boston, MA, USA). Conditions were tested in triplicates or quadruplicates. An unpaired student t-test was performed to compare statistical differences between two treatment groups (spheroids in free media and spheroids pretreated with collagen then degraded with Collagenase D). Values associated with *p* ≤ 0.05 were considered significant.

## 3. Results

### 3.1. Evaluation of FAM-fTHP-9 Signal Detection

We first examined whether the signal emanating from the cleaved FAM-fTHP-9 substrate could be detected by the monochromators of the Cytation 5. In addition, it was also imperative that the cleaved substrate signal be statistically greater than that detected from wells containing no substrate. To this end, U-87 glioblastoma spheroids were used as a test cell line for assay development. FAM-fTHP-9 was added to wells containing U-87 spheroids. No inhibitory compound was added to these wells. In this manner, the maximum amount of MT1-MMP activity should have been generated in the wells, leading to the largest concentration of cleaved substrate and highest possible fluorescent signal being emitted. Negative control wells were also set up where no substrate was added to uninhibited wells containing U-87 tumoroids. The signal from all wells was then quantified every 6 h over a 36-h period.

**Figure 1:**
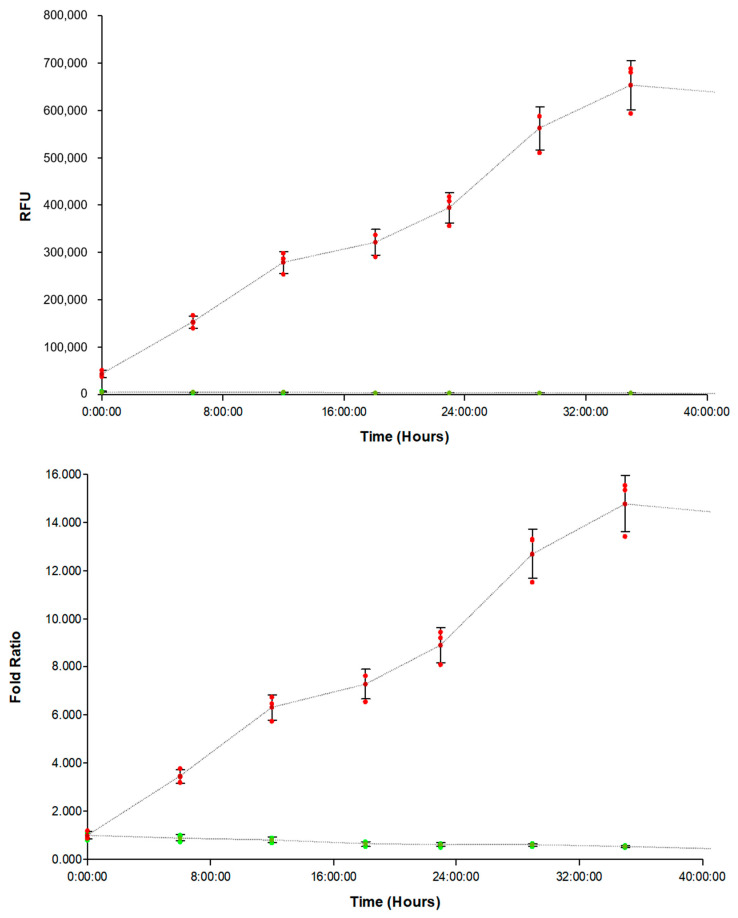
(**Top**) Kinetic FAM-fTHP-9 substrate signal (red) from single uninhibited U-87 test well compared with cells and no substrate (green). (**Bottom**) Ratio of FAM-fTHP-9 substrate signal (red) to background (green) from same uninhibited U-87 test well.

When the average signal from positive control wells, containing substrate, was plotted kinetically over time by the Gen5 software (Figure 1 Top, red data points), the signal could be quantified by the monochromators on the Cytation 5 even when using an embedded 3D spheroid model. In addition, the quantified signal increased over 14-fold over time (Figure 1 Bottom, red data points), as would be expected when incorporating the highly invasive U-87 glioblastoma cell type. When observing the kinetic curve generated from negative control wells, containing no substrate, it was seen that little to no fluorescence was detected at any timepoint during the incubation (Figure 1 Top, green data points). The signal remained stable and did not change in an appreciable manner over time (Figure 1 Bottom, green data points). From the combined results it was clear that the signal from the FAM-fTHP-9 substrate could be accurately detected in a kinetic manner by the Cytation 5′s monochromator system and was statistically greater than background signal.

### 3.2. Correlation of FAM-fTHP-9 Signal with Increased Spheroid Invasion

**Figure 2:**
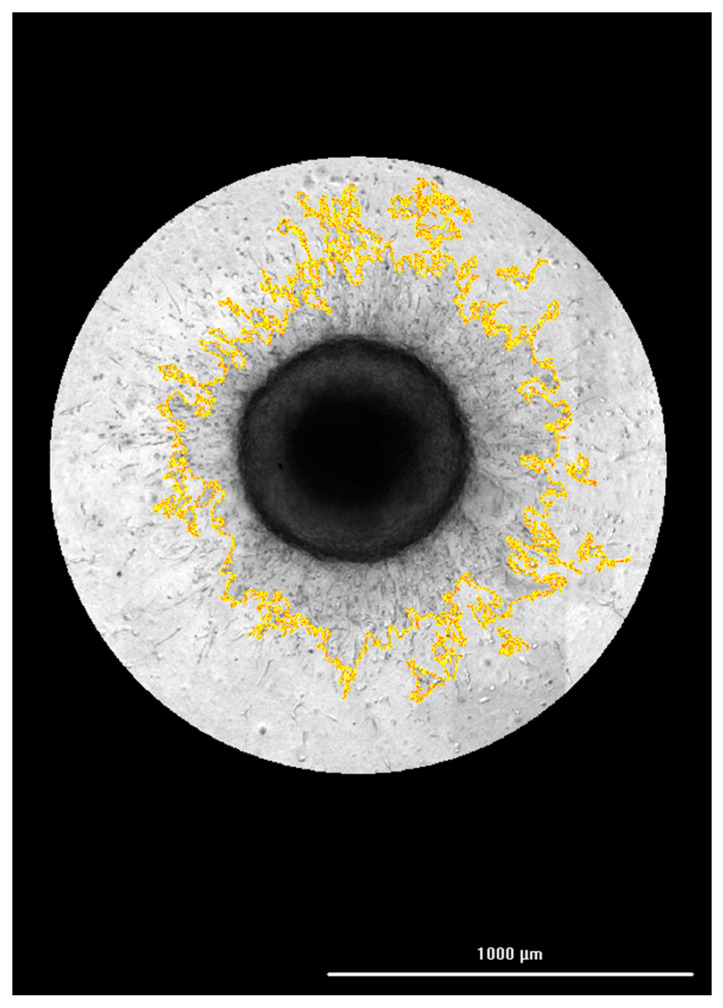
Processed U-87 spheroid invasion image with cellular analysis object mask applied.

In the same experiment where FAM-fTHP-9 substrate signal was being kinetically quantified, brightfield images were captured from wells containing substrate and U-87 spheroids at the same timepoints. Image processing steps were first applied, including stitching, z-projection, and background removal. Cellular analysis was applied to the final image. This allowed a detailed object mask (Figure 2) to be applied to the entire invading structure.

**Figure 3:**
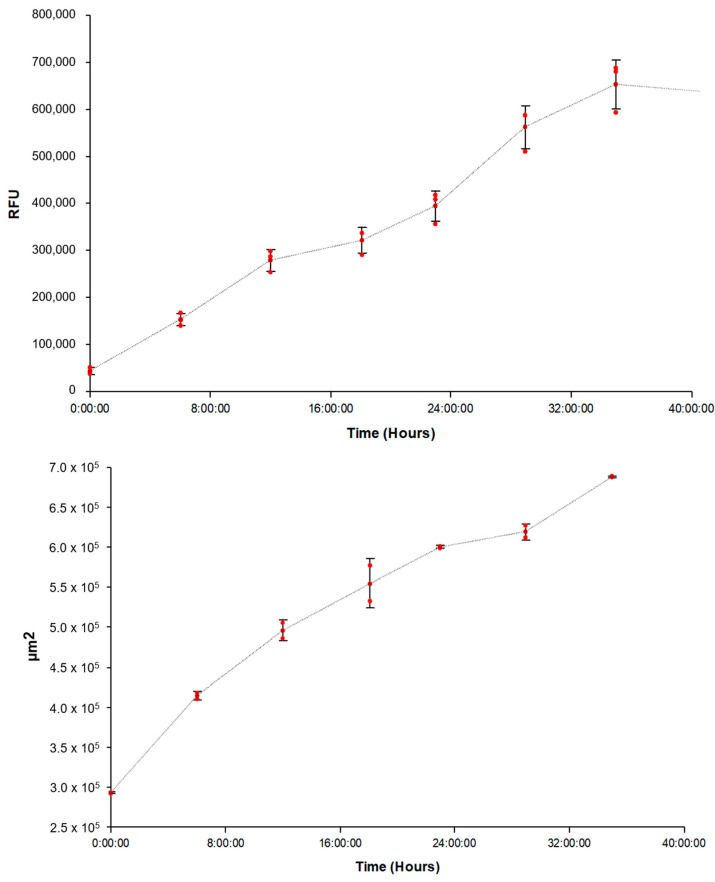
(**Top**) Kinetic FAM-fTHP-9 substrate signal from single uninhibited U-87 test well. (**Bottom**) Kinetic spheroid area coverage from same uninhibited U-87 test well.

When the curve generated by plotting the FAM-fTHP-9 fluorescent signal over time (Figure 3 Top) was compared to the curve created by plotting U-87 spheroid area coverage over the same timeframe (Figure 3 Bottom), it was apparent that an increasing substrate signal is correlated to increasing spheroid invasion into the collagen matrix.

### 3.3. Evaluating the Origin of FAM-fTHP-9 Cleavage

We next evaluated if FAM-fTHP-9 was being cleaved by MT1-MMP and not by other proteases or other means. Marimastat (also known as BB-2516) has an IC_50_ value for MT1-MMP of 1.8 nM [34]. Pro-MT1-MMP can be activated intracellularly by furin and at the cell surface by plasmin [35,36,37]. Aprotinin has an IC_50_ value for plasmin of 21.7 nM [38] but is relatively ineffective against furin [39]. The inhibitors were utilized at concentrations of 400 μM for marimastat and 4 μM for aprotinin to maximize the inhibitory potential. Marimastat and aprotinin were added to designated test wells containing substrate and U-87 spheroids. The wells were then read and imaged kinetically in the same manner as described above, in addition to wells containing uninhibited U-87 spheroids and wells containing spheroids with no substrate. The treatment of spheroids with marimastat indicated that the inhibitor is toxic to cells at a concentration of 1000 μM but not 500 μM and below (Supplementary Figures S1–S3).

**Figure 4:**
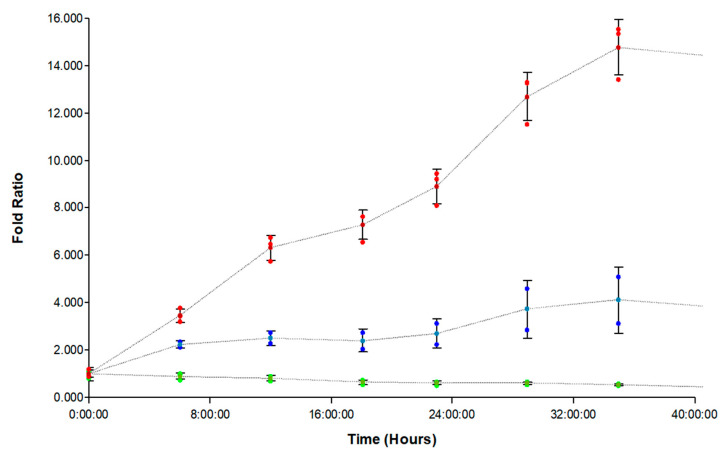
Normalized kinetic substrate signal fold ratio created by dividing signal per timepoint by signal at time 0. Red curve: uninhibited U-87 tumoroids. Blue curve: 400 µM marimastat and 4 µM aprotinin inhibited U-87 tumoroids. Green curve: U-87 tumoroids with no substrate.

When the normalized FAM-fTHP-9 signal was plotted over time, the wells treated with marimastat and aprotinin together demonstrated a significant decrease in substrate signal over the incubation period (Figure 4). This supports the specificity of the substrate, as a decrease in signal indicates diminished substrate cleavage from MT1-MMP enzyme activity due to the inhibitory effects of the two MT1-MMP inhibitors.

In a second experiment, substrate was incubated in the presence of medium alone, and the signal was read over 48 h. Complete DMEM (which included 10% FBS) showed cleavage of FAM-fTHP-9 and that activity was inhibited by PMSF (Supplementary Figure S4), consistent with a prior report [40]. We initially examined cell survival without FBS. Spheroids were viable up to 24 h but not after this (Supplementary Figure S5). A better alternative was to use heat-inactivated media (DMEM with 1% heat-inactivated (HI) FBS). The average fluorescence detected from these wells, throughout the entire incubation period, was 127 RFU, indicating that the medium contained no components that resulted in nonspecific FAM-fTHP-9 cleavage.

### 3.4. Hit Pick Assay Performance Using FAM-fTHP-9 Signal to Trigger Imaging of Test Inhibitor Wells

A Hit Pick experiment was performed to assess the ability of different compounds to inhibit invasion of various spheroids through the collagen matrix. Uninhibited positive control wells were run for each cell type, in addition to the inhibitor containing test wells. A total of 252 wells were included in the experiment across three separate spheroid microplates, including 84 control wells and 168 inhibitor containing test wells. TIMP-2 and NSC405020 have IC_50_ values for MT1-MMP of 5.1 nM and >100 μM, respectively [41,42]. NSC405020 is a non-catalytic inhibitor of MT1-MMP that interacts with the hemopexin-like domain of the enzyme, inhibiting enzyme homodimerization on the cell surface and subsequent cell migration [41]. The inhibitors were utilized at concentrations of 30 μM for TIMP-2 and 100 μM NSC405020 to maximize inhibitory potential.

Following the 48-h incubation period, where cells were in the presence of substrate and inhibitor or no inhibitor, the fluorescent signal was detected from each well of the spheroid plates. The Gen5 software automatically calculated the average signal from the control wells, in addition to standard deviation. A statistically determined Hit Pick criteria for each cell type was established to trigger the imaging of associated test wells. The criteria stated that if the signal from a test well containing inhibitor exhibited a FAM-fTHP-9 signal lower than the average control well signal minus two standard deviations, that test well would be imaged using the brightfield imaging step. If a test well did not meet the criteria described above, then the test molecule did not inhibit substrate cleavage to a statistically significant extent and therefore the well was not imaged. Using the criteria established, out of the total 168 inhibitor containing wells tested, 33 wells were unimaged due to the fact that the substrate signal was not lower than the cutoff value, meaning that MT1-MMP activity was not sufficiently inhibited by the test molecule.

**Figure 5:**
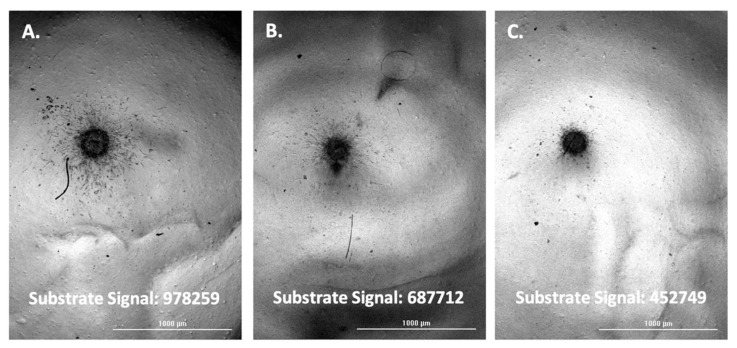
(**A**) A-172 spheroid; uninhibited. (**B**) A-172 spheroid; 30 µM TIMP-2 inhibited. (**C**) A-172 spheroid; 400 µM marimastat, 4 µM aprotinin, 30 µM TIMP-2, 100 µM NSC405020 inhibited.

**Figure 6:**
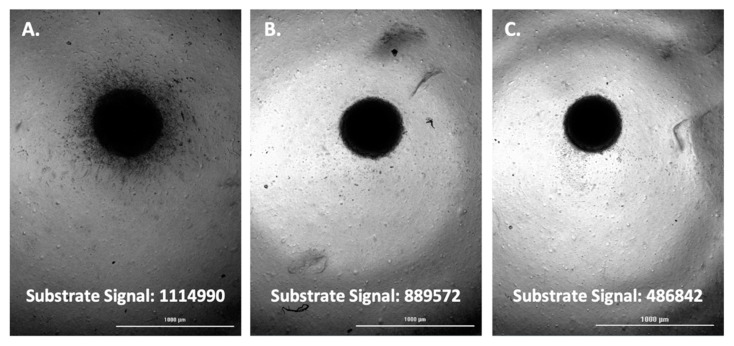
(**A**) H4 spheroid; uninhibited. (**B**) H4 spheroid; marimastat, 400 µM inhibited. (**C**) H4 spheroid; 400 µM marimastat, 4 µM aprotinin, 30 µM TIMP-2, 100 µM NSC405020 inhibited.

**Figure 7:**
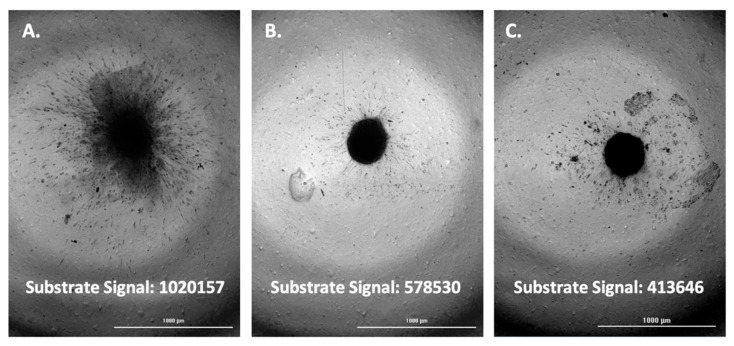
(**A**) SW 1088 spheroid; uninhibited. (**B**) SW 1088 spheroid; marimastat, 400 µM inhibited. (**C**) SW 1088 spheroid; 400 µM marimastat, 4 µM aprotinin, 30 µM TIMP-2, 100 µM NSC405020 inhibited.

**Figure 8:**
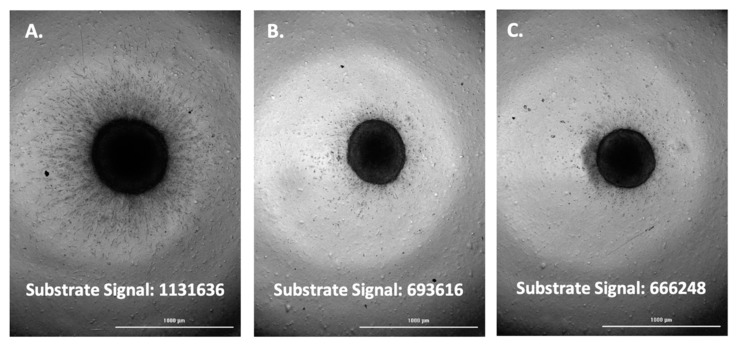
(**A**) U-87 spheroid; uninhibited; substrate signal. (**B**) U-87 spheroid; TIMP-2, 30 µM inhibited. (**C**) U-87 spheroid; 400 µM marimastat, 4 µM aprotinin, 30 µM TIMP-2, 100 µM NSC405020 inhibited.

The fluorescent substrate signal, and associated images, were then compared between positive control wells per cell type and test inhibitor wells triggered to be imaged. This was carried out for each of the four cell types tested; A-172, H4, SW 1088, and U-87 (Figure 5, Figure 6, Figure 7 and Figure 8). 

Upon comparison of the substrate signal with associated images, it was observed that inhibitor wells triggered to be imaged by emitting a lower substrate signal do in fact demonstrate a lower level of spheroid invasion. The results serve to validate the method as a viable way to quickly screen for inhibitors of glioma spheroid invasion.

### 3.5. Evaluation of FAM-fTHP-9 Signal Detection and Spheroid Invasion in A172, H4, and SW 1088 Cell Lines

The hydrolysis of FAM-fTHP-9 was examined for A172, H4, and SW 1088 spheroids. For all three cell lines, increased fluorescence was observed over time (Table 1). A172 spheroids showed the greatest increase in the fluorescence ratio, and hence FAM-fTHP-9 hydrolysis, followed by H4 spheroids and then SW 1088 spheroids. The examination of spheroid growth also showed an increase over time for all three cell lines (Table 1). Spheroid growth did not strictly correlate to an increase in the fluorescence ratio; for example, the A172 spheroids showed the greatest increase in the fluorescence ratio but had a lower growth ratio than the SW 1088 spheroids (Table 1).

### 3.6. Inhibition of FAM-fTHP-9 Signal and Spheroid Invasion in A172, H4, and SW 1088 Cell Lines

The inhibition of the hydrolysis of FAM-fTHP-9 by marimastat plus aprotinin was examined for A172, H4, and SW 1088 spheroids. For all three cell lines, the change in fluorescence over time was decreased when the inhibitors were applied (compare Table 2 and Table 1). The examination of spheroid growth showed virtually no increase over time when the inhibitors were applied (Table 2). The inhibition of spheroid growth for the three cell lines closely resembles that observed in the case of U-87 spheroids (Figure 4).

### 3.7. Western Blot Analysis

Western blot analysis previously demonstrated the presence of MT1-MMP in numerous glioblastoma cell lines [16,43,44,45,46]. During western blot analysis, MT1-MMP can be observed at 69, 63, 57, 53, and 44 kDa, based on glycosylation pattern, proenzyme versus activated enzyme, and autocleavage products [47]. Western blot analysis was performed herein initially for the A172 glioblastoma cell line under various conditions where the protein concentration was normalized to whole cell lysate. MT1-MMP production was below the detection limit in 2D cultures and 3D cultures containing spheroids in media, while spheroids grown in the presence of collagen exhibited the 53 kDa activated enzyme form of MT1-MMP (Supplementary Figure S6). The presence of MT1-MMP was confirmed by comparison to an MT1-MMP standard, as previously demonstrated for pancreatic cancer spheroids [48]. Thus, the 3D culturing of the glioblastoma cell line in collagen resulted in an elevated production of MT1-MMP.

Western blot analysis was repeated using a polyclonal antibody to obtain a stronger signal. MT1-MMP production for the A172 glioblastoma cell line was below the detection limit in 2D cultures, minimal in 3D cultures containing spheroids in media, and significant in spheroids grown in the presence of collagen (Supplementary Figure S7). As observed with the mAb detection, the 3D culturing of the glioblastoma cell line in collagen resulted in an elevated production of MT1-MMP. Conversely, western blot analysis of astrocytes and the SW 1088 astrocytoma cells showed the MT1-MMP production to be substantial in monolayers but below the limits of detection in spheroids (Supplementary Figure S7). The pure MT1-MMP showed bands corresponding to proenzyme (~80 and ~74 kDa), activated enzyme (~53 kDa), and autocleaved enzyme (~33 kDa) (Supplementary Figure S7). The A172 spheroids, SW 1088 monolayers, and astrocyte monolayers primarily showed the ~53 kDa species.

Western blot analysis of melanoma cell lines showed that more MT1-MMP was present in 3D invading WM115 and WM266 spheroids (spheroids contained in collagen) compared to spheroids in media or 2D cell culture (Supplementary Figure S8).

### 3.8. Cell Viability

**Figure 9:**
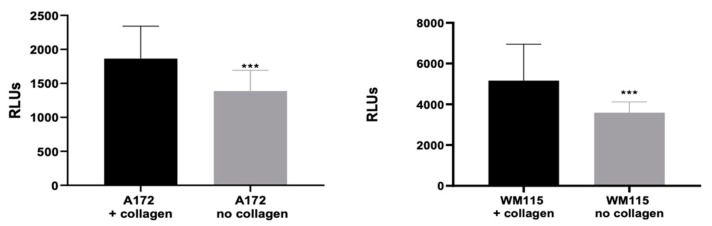
Mean of total relative luminescence units (RLUs) measured in wells containing A172 glioblastoma or WM115 melanoma cells embedded in type I collagen compared to wells without collagen. Statistical analysis was carried out using an unpaired student t-test. *** *p* ≤ 0.0001.

The cell viability assay was based on a luciferase reaction where its co-factor, ATP, was used as an indicator of metabolically active cells and measured using bioluminescence. The initial results revealed very low measurements in terms of relative luminescence units (RLUs) (Supplementary Figure S9), suggesting that either the CellTiter-Glo reagent was unable to penetrate the congealed collagen and reach the spheroids to activate the luciferase reaction or that the 1% acetic acid used to potentially release the spheroids from the collagen was toxic to the cells. Collagenase D was added to the wells of A172 glioblastoma and WM115 melanoma spheroids embedded in type I collagen for the purpose of degrading the collagen in order to obtain accurate results from the cell viability assay. A172 and WM115 spheroids pretreated with collagen followed by an addition of collagenase D resulted in the significant appearance of RLUs (Figure 9). Spheroids embedded in collagen showed an increase in RLUs compared with spheroids not embedded in collagen, indicating improved cell viability in the collagen environment.

## 4. Discussion

MT1-MMP has long been implicated in tumor metastasis and angiogenesis [49]. The development of MT1-MMP inhibitors would be greatly facilitated by their evaluation in a tumor-mimicking microenvironment. Three-dimensional models such as spheroids and organoids provide systems that allow for the rapid screening of novel therapeutics while maintaining cellular behaviors more reminiscent of tumors than traditional 2D cell culture [26,50,51].

Methods to quantitatively assess MT1-MMP activity in a cellular environment have been lacking [49,52]. The visualization of membrane-bound, active MT1-MMP has been achieved by fluorescence resonance energy transfer (FRET) or the bioluminescence imaging of sensors, often surface-anchored, but these methods do not provide insight into MT1-MMP kinetics [53,54,55,56,57,58,59,60,61]. We previously described a 2D cell-based assay for MT1-MMP that allowed for the quantification of enzyme activity and inhibition [52]. The assay utilized a triple-helical FRET substrate, where the combination of sequence and triple-helical structure minimized non-specific proteolysis. We improved on the prior method by changing the fluorophore/quencher pair from (7-methoxycoumarin-4-yl)-acetyl (Mca)/2,4-dinitrophenyl (Dnp) to 5-carboxyfluorescein (5-Fam)/4,4-dimethylamino-azobenzene-4′-carboxylic acid (Dabcyl). The higher wavelength used to monitor 5-Fam fluorescence (λ_excitation_ = 490 nm and λ_emission_ = 520 nm) leads to less background signal from cells. The 3D spheroid model described herein showed that MT1-MMP activity correlated to tumor invasion (Figure 3), and thus the method represents a potentially effective screening tool for MT1-MMP inhibitors. The presence of MT1-MMP in cell lines was confirmed by western blotting (Figure 9), consistent with prior results [16,46]. For example, the U-87 glioblastoma cell line expresses MT1-MMP and produces the enzyme on the cell surface [16,43,44] while BT25 gliobastoma and BT114 glioma spheroids produce MT1-MMP [45].

UWR2 and UWR3 glioblastoma cell lines produced more MT1-MMP compared to SNB-19 astrocytoma [62]. In similar fashion, U-87 glioblastoma cells produced more MT1-MMP compared to IPSB18 anaplastic astrocytoma [16]. Glioblastomas typically have higher MT1-MMP gene expression than astrocytomas, although this is not absolute [16,43,46,62,63]. Based on the present and prior results, U-87 and A-172 glioblastoma cells have higher levels of MT1-MMP compared with astrocytoma cells (H4 and SW 1088 herein). In turn, the levels of MT1-MMP in the spheroids examined correlated to the rate of substrate hydrolysis (Figure 1 and Figure 9 and Table 1), indicating that the assay was effective for monitoring MT1-MMP activity.

The inhibition of MT1-MMP activity resulted in decreased tumor invasion (Figure 4, Figure 5, Figure 6, Figure 7 and Figure 8 and Table 2), indicating a significant role for this enzyme in the metastatic process. Applying MT1-MMP inhibitors, in addition to slowing metastasis, may improve immune responses. MT1-MMP sheds tumor cell MHC class I chain-related molecule A (MICA) [64]. The engagement of MICA to NKG2D stimulates natural killer (NK) and T-cell antitumor activity [64]. The inhibition of MICA and MICB shedding promotes NK cell antitumor immunity [65].

Evaluating the activity of MT1-MMP in the tumor environment can facilitate the application of MT1-MMP activated prodrugs [66]. For example, ICT2588 is an MT1-MMP activated prodrug that delivers colchicine to the tumor vasculature [67,68,69]. More specifically, ICT2588 is composed of (1) azademethylcolchicine at the *C*-terminus, (2) the peptide sequence Arg-Ser-Cit-Gly~Hof-Tyr-Leu-Tyr (where Cit = citrulline and Hof = homophenylalanine), which is cleaved selectively by MT1-MMP at the Gly~Hof bond, and (3) fluorescein at the *N*-terminus [67,68,69]. ICT2588 was subsequently modified by utilizing the sequence βAla-Cys-Arg-Ser-Cit-Gly~Hof-Tyr-Leu-Tyr and attaching cross-linked iron oxide particles to the side chain of Cys [70]. The resultant CLIO-ICT theranostic was utilized for treatment of glioblastoma [71,72,73]. In a similar fashion, the Pro-Cit-Gly~Hof-Tyr-Leu MT1-MMP selective sequence has been used for delivery of a doxorubicin prodrug [74]. A dual near-infrared fluorescence (NIRF)/positron emission tomography (PET) probe, activated by MT1-MMP processing of the Arg-Ser-Cit-Gly~Hof-Tyr-Leu-Tyr sequence, has been used to image glioma in preclinical models [75].

The present screening approach utilized cell line-derived spheroids and thus does not capture a true tumor microenvironment. In turn, the assay appears to be readily amenable to adaptation, so a more tumor-like environment could be incorporated. For example, glioblastoma cells could be seeded in type I collagen or hyaluronic acid hydrogels supplemented with native brain-derived ECM [76]. Including other cell types found in the tumor microenvironment would allow for the examination of indirect effects, such as glioma-associated microglia/macrophages producing MT1-MMP, which then activates glioma-derived proMMP-2 and enhances tumor invasion [10]. It will ultimately be important to better understand the temporal and spatial activity of MT1-MMP in glioma [77]. While glioblastoma is currently categorized as either isocitrate dehydrogenase wild type or isocitrate dehydrogenase mutant, the identification of subtypes within these two categories [46,78,79,80], including levels of MT1-MMP expression, may provide insight into patient-specific treatment strategies.

## 5. Conclusions

FAM-fTHP-9 was found to accurately detect MT1-MMP activity in tumor spheroids. Signal from the cleaved substrate increased over time proportionately to spheroid invasion. The substrate itself was also shown to be specific to cleavage from the target MT1-MMP, with the signal production being reduced accordingly by known MT1-MMP inhibitors. When combined together, the substrate and Hit Pick process provided an efficient, yet robust, method to predict the potential inhibitory effects of test molecules on MT1-MMP activity and downstream tumor invasion in 3D glioma cell models.

## Figures and Tables

**Table 1:** Substrate hydrolysis and spheroid growth.

Cell Type	Time (h)	Substrate Signal (RFU)	Substrate Ratio	Spheroid Area (μm^2^)	Spheroid Ratio
A172	0	4.85 ± 0.13 × 10^4^	1.00	8.22 ± 0.23 × 10^4^	1.00
	24	3.52 ± 0.03 × 10^5^	7.26 ± 0.25	1.35 ± 0.05 × 10^5^	1.64 ± 0.10
	48	5.77 ± 0.05 × 10^5^	11.9 ± 0.22	1.8 ± 0.15 × 10^5^	2.19 ± 0.12
H4	0 24	8.92 ± 0.23 × 10^4^ 5.12 ± 0.15 × 10^5^	1.00 5.72 ± 0.02	2.93 ± 0.05 × 10^5^ 4.53 ± 0.08 × 10^5^	1.00 1.55 ± 0.01
	48	8.62 ± 0.11 × 10^5^	9.66 ± 0.13	6.64 ± 0.02 × 10^5^	2.27 ± 0.05
SW 1088	0	9.9 ± 0.2 × 10^4^	1.00	1.64 ± 0.03 × 10^5^	1.00
	24	4.31 ± 0.32 × 10^5^	4.35 ± 42	3.79 ± 0.17 × 10^5^	2.31 ± 0.14
	48	7.62 ± 0.47 × 10^5^	7.7 ± 0.65	4.84 ± 0.31 × 10^5^	2.96 ± 0.25

**Table 2:** Inhibition of substrate hydrolysis and spheroid growth by marimastat + aprotinin.

Cell Type	Time (h)	Substrate Signal (RFU)	Substrate Ratio	Spheroid Area (μm^2^)	Spheroid Ratio
A172	0	4.80 ± 0.26 × 10^4^	1.00	6.06 ± 0.06 × 10^4^	1.00
	24	1.31 ± 0.03 × 10^5^	2.73 ± 0.2	6.13 ± 0.07 × 10^4^	1.01 ± 0.02
	48	2.0 ± 0.05 × 10^5^	4.17 ± 0.31	5.57 ± 0.54 × 10^4^	0.92 ± 0.1
H4	0 24	8.79 ± 0.42 × 10^4^ 1.33 ± 0.05 × 10^5^	1.00 1.52 ± 0.02	2.28 ± 0.01 × 10^5^ 2.29 ± 0.02 × 10^5^	1.00 1.01 ± 0.01
	48	2.72 ± 0.15 × 10^5^	3.10 ± 0.03	2.3 ± 0.02 × 10^5^	1.01 ± 0.01
SW 1088	0	9.51 ± 0.22 × 10^4^	1.00	1.46 ± 0.05 × 10^5^	1.00
	24	1.1 ± 0.07 × 10^5^	1.16 ± 0.05	1.4 ± 0.1 × 10^5^	0.97 ± 0.04
	48	2.69 ± 0.07 × 10^5^	2.83 ± 0.14	1.34 ± 0.1 × 10^5^	0.92 ± 0.04

## Data Availability

The data presented in this study are available on request from the corresponding author. The data are not publicly available due to a confidentiality agreement between Agilent Technologies and Florida Atlantic University.

## Supplementary Materials

The following supporting information can be downloaded at: https://www.mdpi.com/article/10.3390/biomedicines11020562/s1, Figure S1: Viability of WM266 spheroids in the presence of marimastat over time; Figure S2: Processing of FAM-fTHP-9 by WM266 spheroids in the presence of marimastat after 24 h; Figure S3: Processing of FAM-fTHP-9 by WM266 spheroids in the presence of marimastat after 72 h; Figure S4: DMEM processing of FAM-fTHP-9 and inhibition by no or 1.0 mM PMSF; Figure S5: WM266 spheroid survival in DMEM with and without FBS and phenol red over time; Figure S6: Western blot analysis of A172 cell culture, spheroids, and spheroids incubated with collagen for detection of MT1-MMP using a mAb; Figure S7: Western blot analysis of MT1-MMP; Figure S8: Western blot of WM115 and WM266 cell culture and spheroids for detection of MT1-MMP; Figure S9: Spheroid viability in the presence of 1% acetic acid.
