# Supplementary material for: Screening MT1-MMP Activity and Inhibition in Three-Dimensional Tumor Spheroids
Source: Biomedicines. 2023 Feb 15;11(2):562. doi: 10.3390/biomedicines11020562 (PMC9953393; doi:10.3390/biomedicines11020562)
Supplement: Supplementary file 1 [file biomedicines-11-00562-s001.zip › biomedicines-1910895-supplementary.pdf]

Supporting Information

Screening MT1-MMP Activity and Inhibition in Three-dimensional Tumor Spheroids

Anna M. Knapinska, Gary Drotleff, Cedric Chai, Destiny Twohill, Alexa Ernce, Dorota Tokmina-Roszyk, Isabella Grande, Michelle Rodriguez, Brad Larson and Gregg B. Fields

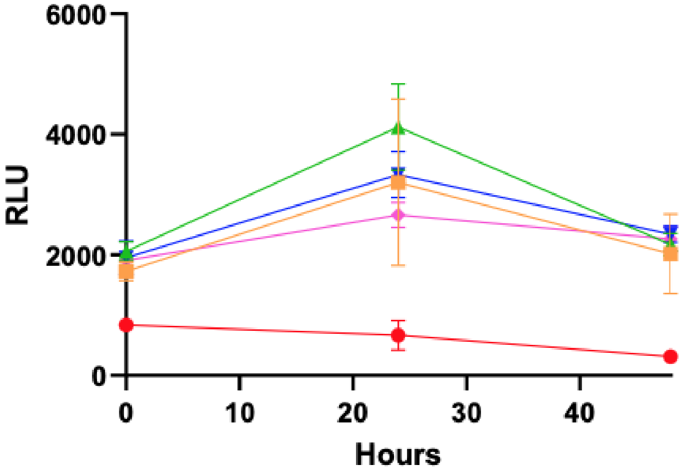

Figure S1: Viability of WM266 spheroids in the presence of 0 (purple diamond), 125 (blue inverted triangle), 250 (green triangle), 500 (orange square), and 1000 (red circle) μM marimastat over time.

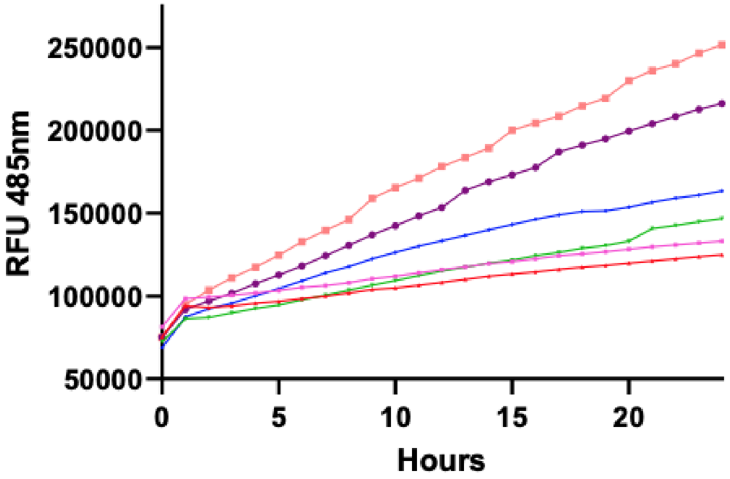

Figure S2: Processing of FAM-fTHP-9 by WM266 spheroids in the presence of 3 (pink square), 131 (purple circle), 197 (blue diamond), 296 (green inverted triangle), 444 (red diamond), and 667 (purple square) μM marimastat after 24 h.

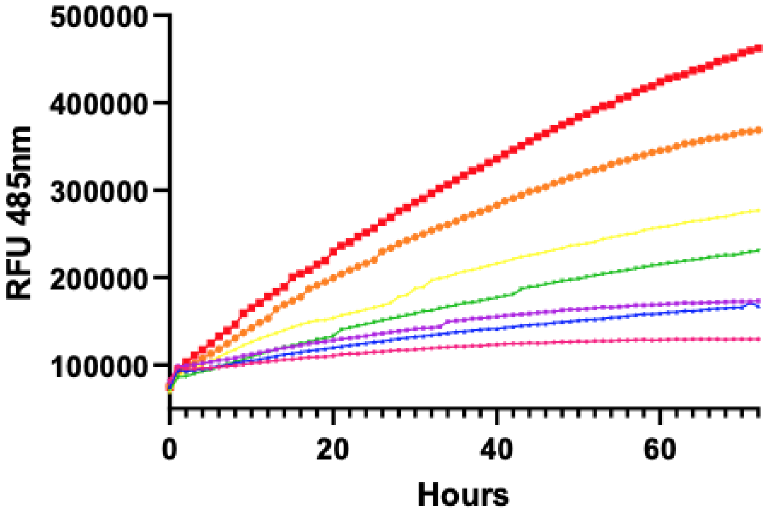

Figure S3: Processing of FAM-fTHP-9 by WM266 spheroids in the presence of 3 (red square), 131 (orange diamond), 197 (yellow square), 296 (green inverted triangle), 444 (blue triangle), 667 (purple square), and 1000 (pink circle) μM marimastat after 72 h.

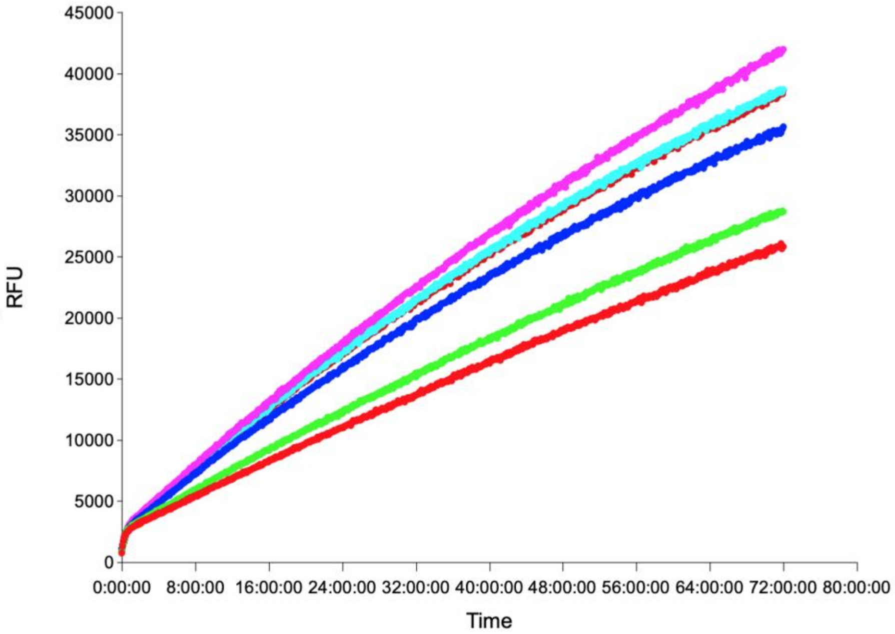

Figure S4: DMEM processing of FAM-fTHP-9 and inhibition by no (dark red, purple, and aqua) or 1.0 mM (blue, green, and red) PMSF.

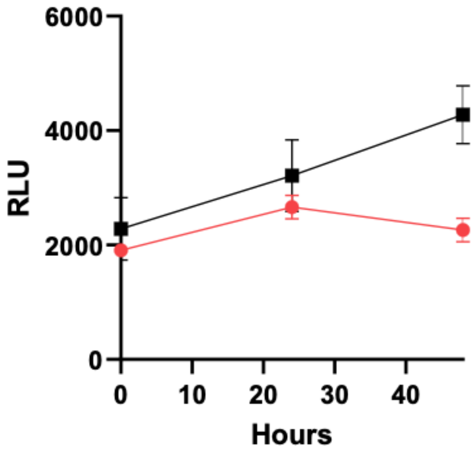

Figure S5: WM266 spheroid survival in DMEM with (black squares) and without (red circles) FBS and phenol red over time.

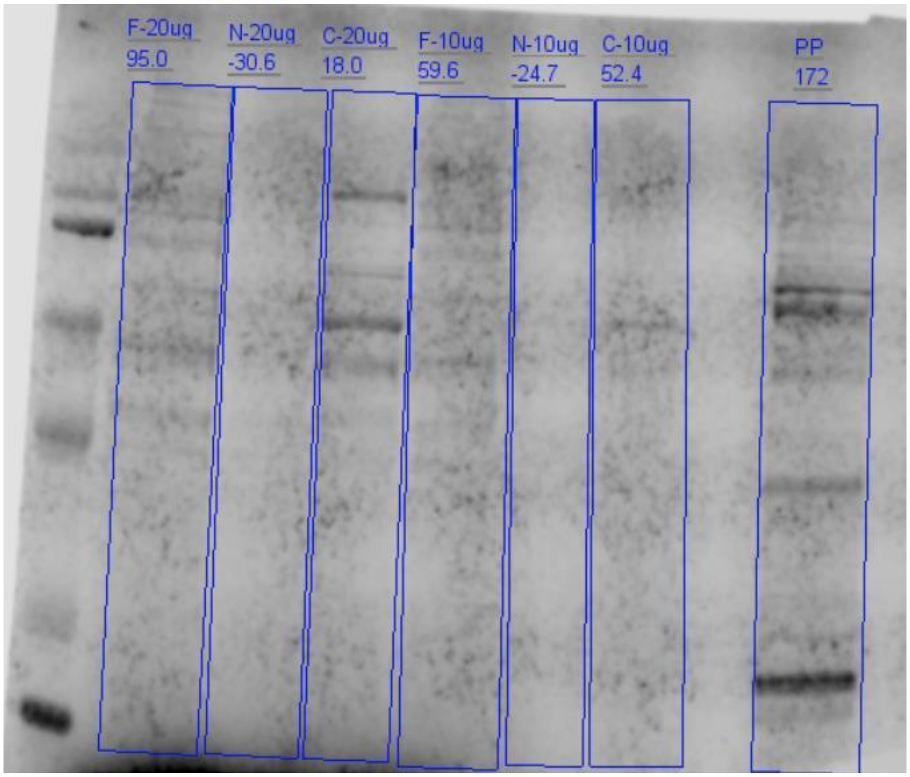

Figure S6: Western blot analysis of A172 cell culture (F), spheroids (N), and spheroids incubated with collagen (1:3) for detection of MT1-MMP using a mAb. Gel shows 53 kDa MT1-MMP when collagen is present, while pure MT1-MMP (PP) shows the proenzyme and 33 kDa autocleavage product.

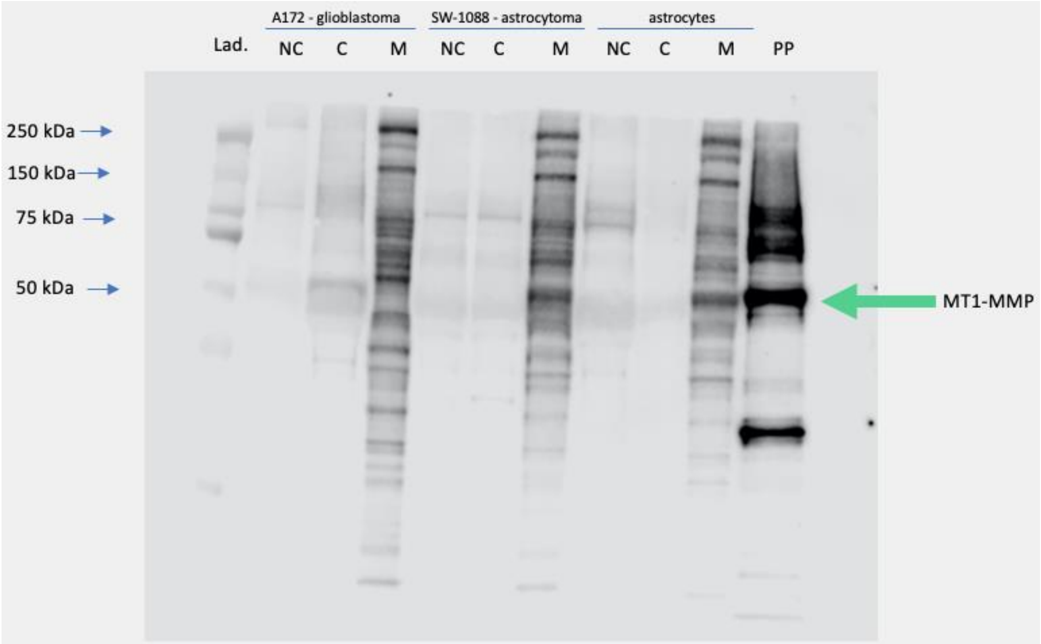

Figure S7: Western blot analysis of MT1-MMP. NC = No collagen spheroid, C = Collagen spheroid, M = Monolayer cell culture, Lad = Ladder with standards, PP = Pure protein. For each sample 20 μg of protein was loaded.

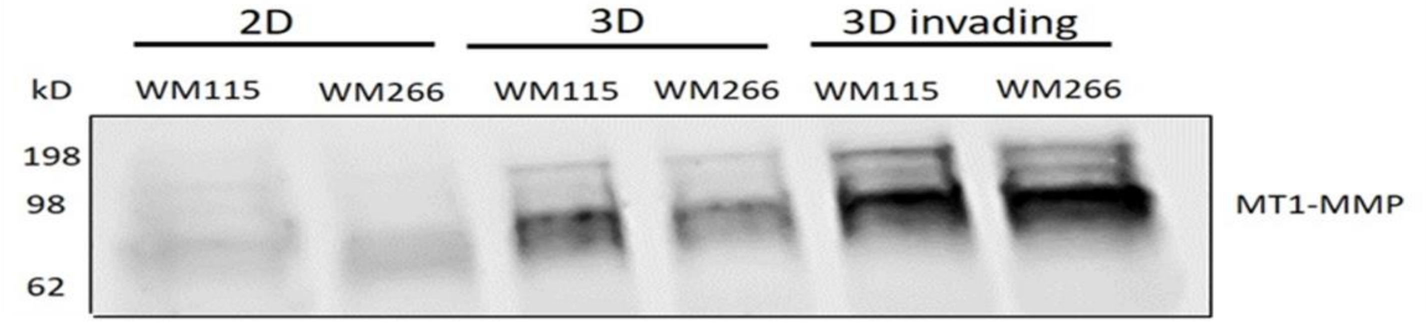

Figure S8: Western blot of WM115 and WM266 cell culture (2D) and spheroids (3D) for detection of MT1-MMP.

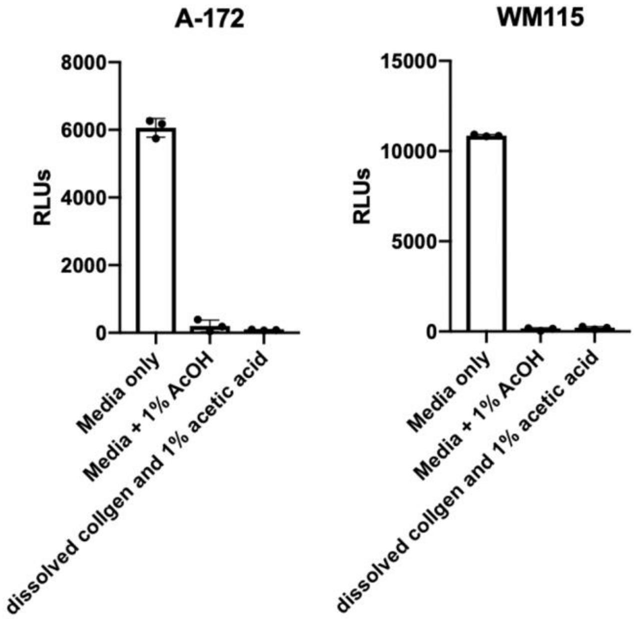

Figure S9: Spheroid viability in the presence of 1% acetic acid.
